# Anything that helps: cancer patient and carer perspectives on psychedelic-assisted therapy

**DOI:** 10.1007/s00520-026-10693-z

**Published:** 2026-05-04

**Authors:** A. Wech, A. Akroyd, C. Clayden, L. M. Reynolds

**Affiliations:** Department of Psychological Medicine, Faculty of Medical and Health Sciences, University of Auckland, 22-30 Park Avenue, Grafton, Auckland, 1023 New Zealand

**Keywords:** Psychedelic-assisted therapy, PAT, Cancer, Caregivers, Psychedelics, Cancer-related distress

## Abstract

**Purpose:**

The potential of psychedelic-assisted therapies (PAT) to address existential distress in cancer populations is attracting increasing attention. However, this novel approach is challenged by stigma, hype, and misconceptions. The current study aimed to investigate the knowledge, beliefs, and attitudes of advanced cancer patients and carers regarding psychedelics and their potential therapeutic use in treating cancer-related distress.

**Methods:**

Fifteen semi-structured interviews were conducted with cancer patients and carers. Participants needed to be at least 18 years old and either diagnosed with stage 4 cancer and have mild to moderate distress as measured by the General Anxiety Disorder 7-Item Scale and Patient Health Questionnaire 9, or be caring for someone with advanced cancer. Data were analysed using reflexive thematic analysis.

**Results:**

Primary themes centred around how the perspective of cancer patients and their carers changes at the end of life, and the importance of balancing safety and risk with potential benefits.

Participants expressed largely positive views of PAT, recognising that with few available options and often poor quality of life, any intervention offering potential benefit was worthwhile. However, acceptance of PAT was tempered by a desire to minimise risk and concerns about safety.

**Conclusion:**

Our findings demonstrate that cancer patients and their carers are open to the idea of psychedelic therapies as long as risk is carefully managed. It is important that the perspectives of patients and carers are included in developing PAT interventions given their potential to offer a meaningful option in improving the lives of those with advanced cancer.

## Purpose

Advanced cancer often entails profound physical, psychological, emotional, and spiritual challenges that can produce clinical levels of anxiety and depression in patients [1]. As cancer advances, both awareness of prognosis and symptomatic burden grow, leaving patients with late-stage or untreatable cancers at greatest risk of psychological distress [2, 3]. Research suggests death anxiety is also common in advanced cancer with reports of up to 32% of patients experiencing moderate to significant levels of death anxiety [4]. Similarly, informal carers of people with advanced cancer report high levels of anxiety and depression [5]. Despite evidence demonstrating the prevalence of distress in this population, it remains poorly managed by conventional approaches. Empirically supported interventions for anxiety and depression such as cognitive-behavioural therapy (CBT) [6], and pharmaceutical treatments [7] are not always effective in this patient group.

An emerging treatment that may hold promise in advanced cancer is administering psychedelic compounds alongside psychotherapy. Evidence suggests psychedelics can be effective in improving treatment-resistant depression and anxiety [8, 9], notably when paired with psychotherapy [10, 11]. The mechanisms of effect are still unclear but may involve the psychedelic experience facilitating meaning making [12], changes in neuroplasticity [13], feelings of connectedness, and reduced fears of mortality [14].

Though constituting a small body of research, findings from studies using psychedelic-assisted therapies (PAT) with cancer patients support their theorised efficacy. Reductions in anxiety have been noted in people with life-limiting diseases following LSD-assisted psychotherapy [15], and studies specifically with cancer patients have found improvements in emotional processing of existential distress [16], anxiety, depression, and quality of life [17–20]. Long-term follow-up with participants has indicated sustained benefits, with reductions in anxiety and depression remaining at 5-year follow-up [21]. 

Despite offering promise as a psychotherapeutic adjunct, the use of psychedelics with advanced-cancer patients faces various likely barriers. Psychedelics are largely classified as Schedule 1 substances, meaning they are assessed as entailing a very high risk of harm. Additionally, stigma related to “hippie” culture of the ‘60 s and ‘70 s may entail negative beliefs. Negative framing within discourse of mainstream media also likely informs concerns about their use. In combination with the relative vulnerability of advanced-cancer patients, it seems reasonable to assume this treatment approach may garner apprehension. However, conversely, there has also been recent discourse about psychedelics as a panacea, potentially urging the acceptance of use in psychological treatments [22, 23]. A recent systematic review of the perceptions and attitudes towards psychedelic-assisted psychotherapy among health professionals, patients, and the public [24] found knowledge of such therapies was low across all groups and, importantly, that greater awareness was associated with more positive beliefs. The majority of studies in this review reported strong endorsement of further research. However, there has been minimal research specifically exploring the perspectives of advanced-cancer patients and their carers. The present study aimed to investigate the knowledge, beliefs, and attitudes of advanced-cancer patients and carers regarding psychedelics and their therapeutic use in treating cancer-related distress.

## Methods

A qualitative approach was chosen to assess the views of advanced cancer patients and carers in an exploratory and unbounded manner. Fifteen semi-structured interviews were conducted, and data were analysed using reflexive thematic analysis. A realist/essentialist paradigm was employed, conceptualising language as a literal reflection of experience and reality. The researchers were a mix of academic and health psychology clinicians and, as such, brought primarily biomedical and psychological perspectives to this work. The analysis primarily described participants’ perceptions, beliefs, and experiences, rather than critically examining how these were constructed. As such, the findings reflect participants’ reported experiences and perspectives.

### Participants

Participants were either advanced cancer patients or their carers. Eligibility required all individuals to be at least 18 years old and either 1) diagnosed with stage 4 cancer and have mild to moderate distress as measured by scores between 5 and 19 on both the General Anxiety Disorder 7-Item Scale (GAD-7) and Patient Health Questionnaire 9 (PHQ-9) or 2) be caring for someone with advanced cancer.

### Study procedure

Participants were recruited using advertisements via cancer support organisations, referrals from public health services, or opportunistic recruitment with existing participants who discussed the opportunity with associates. Study flyers asked for volunteers who have advanced cancer and are feeling down or anxious, or are caring for someone who is. To minimise the possibility of a sample bias, flyers did not mention psychedelics and simply asked “can you help us with our research about advanced cancer”. A total of 21 prospective patients/carers were screened for eligibility. Screening assessed demographic details, basic information about diagnosis, treatment and relevant medical history in patients, and patients were additionally assessed for psychological distress as measured by GAD-7 and PHQ-9.

**Fig. 1:**
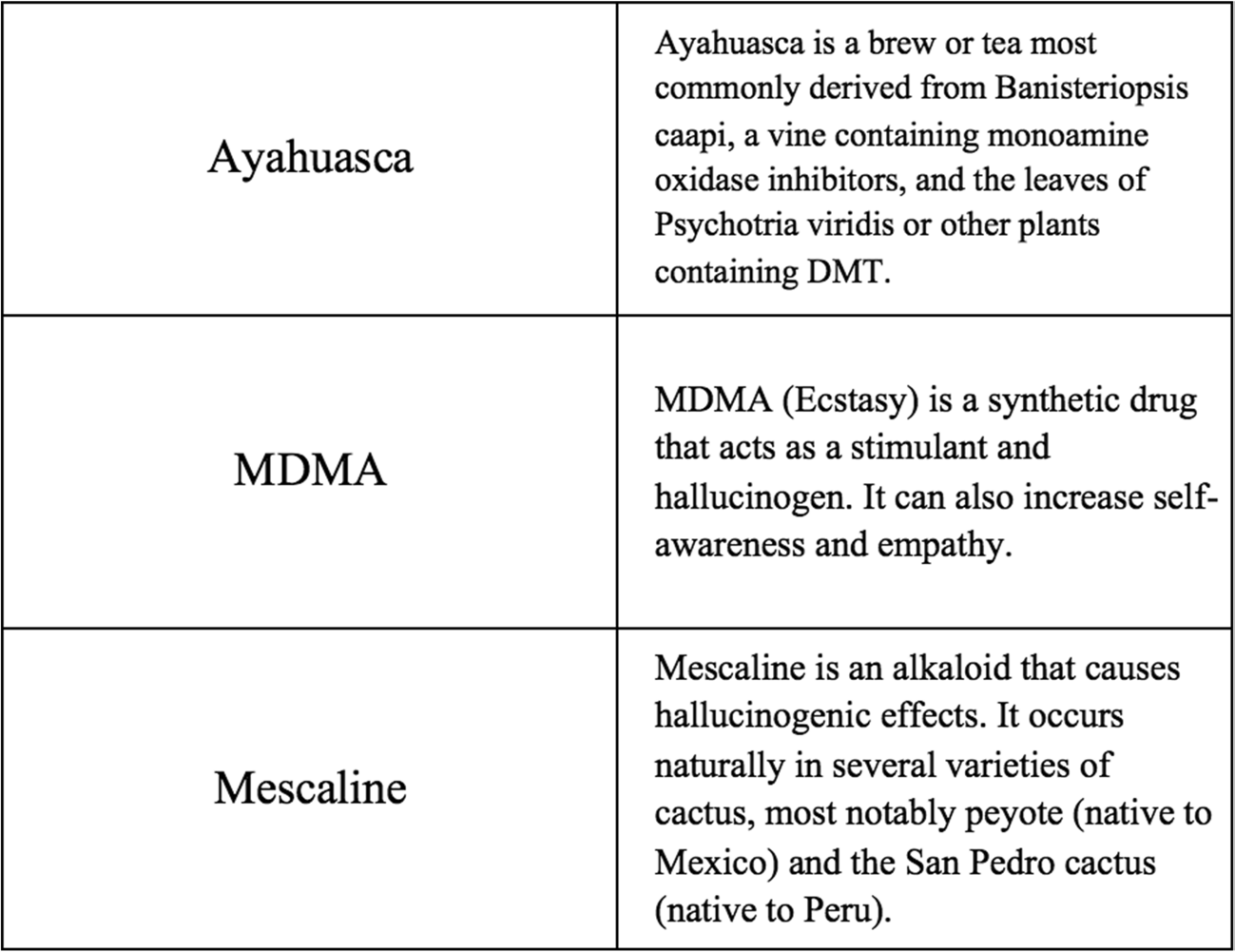
Example of informational flash cards shown to participants

Semi-structured interviews were guided by a preformulated interview guide which asked about participants’ knowledge and perceptions regarding psychedelic compounds generally, as well as their specific therapeutic use with cancer patients. Questions included “what does the term psychedelics bring to mind?” and “what are your feelings towards psychedelics?” For in-person interviews, flash cards with relevant terms and their definitions were used to stimulate conversation about participants’ knowledge and reactions to specific concepts; this was adapted for verbal delivery in video-conference/phone interviews (see Fig. 1 for example).

As part of the interview, participants were presented with the following summary of findings from Ross et al. [18] and Griffiths et al. [17]:

A couple of recent clinical trials have investigated the use of a single high dose of Psilocybin alongside psychotherapy in people with advanced cancer. Participants reported clinically significant improvements in anxiety, depression, and overall quality of life. These effects were sustained in 60–80% of participants at 6 months follow up.

All interviews were recorded using either a Sony ICDPX470 Digital Voice Recorder or the record function of video-conference software.

All interviews were transcribed verbatim by researchers AA and AW. Transcripts were compiled using NVivo 12 by AW. Initial coding and organising of themes were completed manually by AW and were iteratively reviewed and revised in consultation with LR and CC. Reflexive thematic analysis was used following the steps outlined by Braun and Clarke [25]. A large number of codes were initially produced and these were refined into potential themes. The final stages of analysis involved piecing together themes in relation to the underlying narrative, as well as within the context of wider literature.

## Findings

Demographic data and diagnostic information about participants are displayed in Table 1. Most participants were female, Pākehā/New Zealand European, and ranged in age from 20 to 75 years.

**Table 1:** Demographic information and diagnosis for individual participants

*Participant	Gender	Age	Ethnicity	Diagnosis
Paul (C)	M	55–60	Pākehā/NZE	N/A
Marama (P)	F	55–60	Māori	Breast cancer
Pania (C)	F	25–30	Māori	N/A
Josh (C)	M	20–25	Pākehā/NZE	N/A
Anna (P)	F	45–50	Pākehā/NZE	Breast cancer
Yvonne (P)	F	70–75	Pākehā/NZE	Thyroid cancer
Chris (C)	M	45–50	Australian	N/A
Michael (P)	M	85–90	Pākehā/NZE	Kidney cancer
Kim (P)	F	55–60	Pākehā/NZE	Acute myeloid leukaemia
Margaret (P)	F	70–75	Pākehā/NZE	Breast cancer
Paula (P)	F	50–55	Canadian	Malignant glioma
Ellen (P)	F	55–60	Pākehā/NZE	Breast cancer
Yitong (P)	F	65–70	Asian	Ovarian (stage unspecified)
Elizabeth (P)	F	55–60	Pākehā/NZE	Breast cancer
Sarah (P)	F	60–65	Pākehā/NZE	Endometrial cancer

Diagnoses are given in terms as reported by participants

Participant pseudonyms are presented with their categorisation as either a carer (C) or a patient (P)

*M* male, *F* female, *NZE* New Zealand European

### Themes

**Fig. 2:**
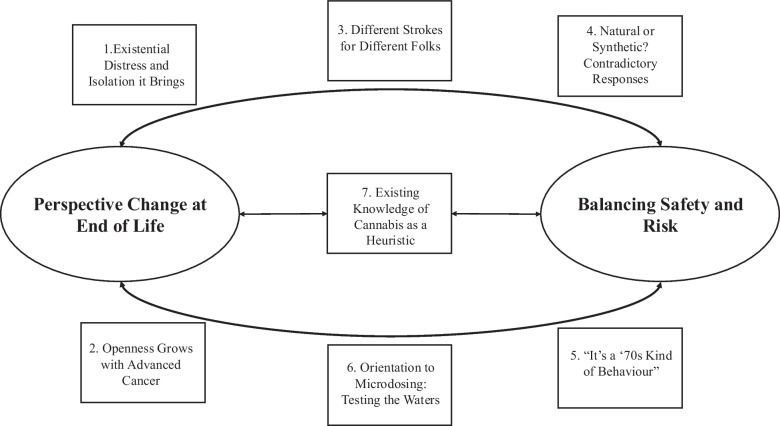
Schematic showing organisation of seven identified sub-themes into two higher-level themes. Note. Theme titles are abbreviated in places for formatting

Two main themes and seven sub-themes emerged from participant responses. The interrelated sub-themes and main themes are illustrated in Fig. 2 (see Table 2 for representative participant quotes of sub-themes).

**Table 2:** Representative participant quotes of sub-themess

Existential distress and the isolation it brings
“they have that constant existential crisis going on. Am I going to make it? If I die what’s on the other side? I’m going to be leaving my friends and family behind.” (Chris, 47, C)
“you’re basically just being handed down a death sentence that you’ve got to deal with when you’re alive, and then you’ve got to try move on with living” (Anna, 46, P)
“The how long have I got to live, how long have I got to live, how long have I got to live, I just wanted to know” (Elizabeth, 57, P)
“nobody knows what it’s like except somebody else who’s that position” (Elizabeth, 57, P)
“Anything that helps”: Openness grows in the face of advanced cancer
“I have positive thoughts about them [psychedelic therapies] coming through if they can bring them through to help people, it’s just great” (Marama, 56, P)
“I’m all for anything that makes people less anxious or happier, or less pain.” (Yvonne, 71, P)
“Yeah I mean look it’s the benefits are quite plain to see you know I think it’s phenomenal to just be able to have that one session like that and to be able to get sustained long term benefit out of it for someone to help them get rid of all that anxiety, it’s fantastic, yeah.” (Chris, 47, C)
“I think the thing about being at Stage 4 is that unless you’re one of those unlucky people who discovers they’re Stage 4 when they first discover they have cancer, which happens to some people, there’s normally a bunch of chemo and other nasty shit that you’ve done before you’ve got to the Stage 4 point. So if it’s not a sustained negative, then so what if you maybe feel a bit rough for a few days or something? It’s like, easy. If the benefit is there.” (Elizabeth, 57, P)
“if it can help with somebody through this better, even if it’s only one or two people, then it’s still worth it” (Anna, 46, P)
“I think it’s really good, you know the anxiety and panic attacks that I’ve experienced I wouldn’t wish them on anybody, so if it helps, I would be very open to it, yeah”. (Sarah, 63, P)
Different strokes for different folks
“I think you just need to, try whatever works – you have to try different things to find out what works for you. It’s the thing, and some people will want to try it, and some people just won’t – they won’t feel comfortable with it. Yeah, I think it’s – even the different types of treatment you get for cancer, it varies from person to person. What works for some people doesn’t work for others.” (Anna, 46, P)
“It’s very subjective and open ended really isn’t it, because it would affect everyone differently. No one thing – you can’t look at a group of people and say this is going to work with a group of people, you’ve got to go individually I think.” (Kim, 58, P)
“Yes I’m sure that the more conservative demographic probably would be a bit weary of it. People used to call them square in the 70 s, people who have maybe lived a careful life” (Sarah, 63, P)
“a lot of hard, crusty type of personalities I believe would have difficulty with it” (Michael, 86, P)
“good for some, maybe not for others… but for some, definitely!” (Anna, 46, P)
Natural or Synthetic? Contradictory responses with a common goal of ‘Safety’
“More inclined to take something natural, and that the pharmacist put together.” (Yvonne, 71, P)
“your first instinct is oh well if it’s from a natural source then surely it must be better than something that’s man made, but in reality I’m not sure if that’s true or not”. (Anna, 46, P)
“I tend to lean towards the natural products. But, then again, I don’t know anything about them you see, so I really can’t comment too much on that” (Michael, 86, P)
“[natural substances are] completely harmless, non-toxic, harvested from a mushroom or bark from a tree…[utilising them would] be fantastic, I’d be behind it. I mean, how can you be against someone eating a mushroom, or drinking a mushroom tea, it’s growing in the ground for goodness sake”. (Chris, 47, C)
“It’s a 70’s kind of behaviour”: The past informs current perceptions
“Yeah, probably way back in the 70’s, psychedelic music, you know, and you think of bright colours and flashing lights and that sort of thing.” (Yvonne, 71, P)
“It’s a lot of the 1960 s stuff from Woodstock really isn’t it?” (Kim, 58, P)
“experience around my siblings who grew up in the ‘60 s, from that psychedelics sort of Woodstock era” (Marama, 56, P)
“when I think LSD I think of, you know people in the ‘60 s taking LSD and tripping, and you hear terrible stories about people who took LSD and never came out of the trip”. (Anna, 46, P)
An orientation to microdosing: Testing the waters with psychedelics
“I always say just start out with a small dose, keep working your way up.” (Marama, 56, P)
“I’d like to start off small and then see how it goes, it depends what sort of an effect it’s supposed to have and whether it affects me too much” (Kim, 58, P)
“I mean it’s less risky, in my perception, the microdose” (Elizabeth, 57, P)
“that where a microdose is going to be involved it could be a pretty high powered drug. And if the microdose is appropriate, I would prefer something like that” (Michael, 86, P)
“well I mean given the results of the high dose therapy I would be leaning more in that direction, just sort of the one and done sort of thing”. (Chris, 47, C)
Existing knowledge of cannabis as a heuristic
“It’s like how they’re talking now about CBD and how marijuana was like an illegal – you’re in prison or whatever, and now the push or the flow of that particular herb, [it] is now becoming apparent it’s helping a lot of people with, seizures and things like that.” (Marama, 56, P)
“I know that can be good. I know people who used to smoke marijuana so they could go to sleep so because it’s a natural thing and because it’s in a research control environment that sounds positive and interesting.” (Elizabeth, 57, P)
“I mean, this is a different thing but CBD oil is supposed to be really really good, you know I have a lot of trouble sleeping […] but I know one person in our group is taking it and she says she’s done away with 4 pain killers and she’s sleeping better” (Yvonne, 71, P)
“So I mean my own experience with that kind of thing, not magic mushrooms in particular but I have smoked marijuana before, and the – it is good, but on the flip side also it can be bad” (Anna, 46, P)

*NOTE* Brackets indicate participant pseudonym, age, carer (C) or patient (P)

### Perspective changes at end of life

Several of the sub-themes described how perspectives at the end of life can change, impacting how participants reacted to the concept of PAT.

#### Existential distress and the isolation it brings

To make sense of the way participants responded to the concept of PAT, it is important to consider the broader context within which advanced cancer patients and their carers live**.** Patients and carers often relayed their lived experiences with cancer. Dominant throughout were stories that described profound existential turmoil and the isolation it brings.

Patients paralleled the experience of an advanced cancer diagnosis with capital punishment, with the worry of when life might end being described as “constant”. Here, participants not only communicated anguish tied to receiving a life-threatening diagnosis but also described the challenge for patients in living with this knowledge. Such concerns were described as ever-present. The burden of relentless existential worry was also a factor for carers, who noted the impact on their loved ones of having “this cloud hanging over them” (Chris, 47, C). The other aspect of this theme was that of isolation in feeling this worry. Patients, in particular, frequently described living with advanced cancer as something they felt others could not understand. Secondary-stage or advanced-stage cancer diagnoses were explained by participants as unique and a “very different feeling” (Elizabeth, 57, P). Feelings of existential distress and isolation described by participants here speak to elements of advanced cancer which create a context of vulnerability and desperation; elements which impact the reactions of participants to the concept of PAT.

#### Openness grows with advanced cancer

One of the most consistent themes across the study was the positive reaction to recent PAT research findings. These ranged from intrigue, surprise, and disbelief, to relief at the promise the results hold for advanced-cancer patients. Participants framed their support by acknowledging that options and time are limited, quality of life can be poor, and anything that might help would be valued. Paula (51, P) remarked that “anything that helps” should be used, “even if it was just for half an hour relief, because having cancer is all consuming”. Participants drew on their own experiences of suffering to explain their openness to the concept of PAT. Both patients and carers acknowledged that PAT might carry risk but that the context of advanced cancer is different; as long as an intervention offers some degree of benefit, it is worth trying. Yvonne (71, P) expressed “Yeah, I mean if you’re going to die anyway, it doesn’t really matter if your brain gets a bit altered”. Overall, participants were highly receptive to the concept of PAT and referred to the life-limiting context of advanced cancer in explaining their support.

### Balancing safety and risk

Other sub-themes reflected a desire to balance the risks of novel treatments with a reverence for safety and a desire to avoid further harm.

#### Different strokes for different folks

While many reported support for PAT, others cautioned that one size does not fit all in treatment approaches. Participants considered that personal circumstances vary and some situations might fit better with the use of psychedelics such as where people have existing psychological issues or have not experienced improvement with standard approaches. Others reflected on the type of people that might be opposed to the idea of using psychedelics in this way. Conservatism in particular was cited as an attitudinal barrier by several participants. For instance, those high in religiosity or low in flexibility were expected to have a “focused aspect on how life should be and don’t see any other way” (Marama, 56, P), and would therefore be less open to PAT.

#### Natural or synthetic? Contradictory responses

Discussion about the source of psychedelics—whether it was naturally occurring or manmade—arose spontaneously. Participants’ judgements regarding the source of psychedelic compounds were often contradictory. Nearly all reported a preference for naturally occurring substances, with automatic reactions like “that’s natural, that’s good” (Elizabeth, 57, P) and describing naturally occurring substances as “benign”. However, the same participants often went on to endorse the clinical safety that synthetic medicines bring. The preference for naturally occurring psychedelic compounds was described as largely intuitive, rather than strongly reasoned.

#### “It’s a 70’s kind of behaviour”

A significant theme in participants’ initial reactions was an association between psychedelics and ‘60 s and ‘70 s Hippie culture. All but one participant cited this era or related imagery in their immediate response to the term ‘psychedelics’. Oftentimes, associations to the ‘60 s and ‘70 s were imbued with negative meaning such as experimental drug use and negative reactions*.* Overall, participants reported that memories and cultural understandings of the hippie era played a large role in informing their knowledge. This also appeared to have impacted awareness of specific compounds, such that substances with a greater association to the psychedelic era (i.e. LSD, magic mushrooms) appeared in discussion more commonly than others.

#### Orientation to microdosing: Testing the Waters

Responses during discussion about different approaches to dosing largely suggested a preference for smaller doses, i.e. the idea of microdosing. Patients and carers indicated that being able to test the waters by taking the minimum amount of a compound possible was favourable. For some, a conservative approach to dosing imbued greater safety*.* Toxicity and biological harm were seen as more likely with higher doses. Lower doses were described as less disruptive to the body and “easier for people to cope with” (Margaret, 70, P). One participant described a preference for the higher doses because of the presented research results and because of the convenience it offered in a practical sense. Most participants expressed that the minimal amount of a substance that can produce beneficial effects should be used, a clear reflection of a tension between risk and safety.

#### Existing knowledge of cannabis as a heuristic

Reference to the medicinal use of cannabis was a strong theme in participants’ talk about psychedelics and PAT. At the time of interviews, a referendum on cannabis legislation was being conducted and this likely impacted the strength of this association. Beyond the salience of the public discourseat the time, it also appeared that medicinal marijuana and CBD products provided a frame of reference for moving a mind-altering substance out of the realm of prohibition to a therapeutic treatment. Many described the growing acceptance of cannabis when answering questions about psychedelics. While discourse on cannabis use was referenced to rationalise support for the medicinal potential of psychedelics, some also cited historical experience or knowledge of using cannabis that was negative as the basis for concerns about hallucinogenic compounds. Thus, existing knowledge about cannabis appeared to constitute an essential frame of reference in considering both the positive and negative aspects of psychedelics and PAT.

## Discussion

This study reported largely positive responses from patients and their carers reacting to PAT and the possible development of psychedelic-assisted therapies for cancer-related psychological distress but also noted the importance of safety considerations.

### Perspective changes with advanced cancer

Theme 1 identified descriptions of patient experiences of isolation and existential distress. That participants would describe such fears is unsurprising given that the literature on psychopathology in advanced cancer describes existential distress as increasingly common with disease progression [26]. This theme speaks to the significance of existential distress as a common psychological burden faced by cancer patients.

The intersection between existential distress and isolation has been recognised as a qualitatively distinct form of isolation termed existential loneliness; a feeling of acute awareness that one is separated from others and the universe, and the negative emotion this brings [27]. Such sentiments were reported by participants who felt that no one in their lives could understand their experience. Acknowledgement of existential distress and isolation in the advanced cancer experience is critical to understanding how cancer patients might respond to psychedelic-assisted therapy. Not only does it highlight the necessity for intervention but also reflects a life landscape characterised by vulnerability and desperation, thereby giving context to the reasons why stakeholders in advanced cancer treatments may express a high degree of receptivity to controversial treatments. Additionally, because research suggests that psychedelic experiences might be particularly beneficial for improving feelings of connectedness and reducing death-related anxiety, the presence of existential loneliness in this patient group supports the logic which suggests PAT may be well-suited to use with advanced cancer patients.

Participants framed their support for PAT by acknowledging that options and time are limited, quality of life can be poor, and “anything that helps” has value. This fits with other research demonstrating greater support in situations of unmet need [28] and a need for interventions that alleviate the suffering of cancer patients [29]. However, such sentiments communicate a sense of desperation. Research investigating the use of unconventional therapies in cancer patients has reported feelings of desperation as a common form of motivation to try alternative treatments [30]. It is important to consider that while openness may, at face value, appear positive, receptivity driven by desperation needs to be handled ethically and carefully.

The notion that one size does not fit all informed reactions of both hesitancy and receptiveness to psychedelics. Participants’ reference to the needs of advanced cancer patients was highly individualised, calling for unique treatment approaches on a person-to-person basis. Other qualitative research has reported similar themes, finding that cancer patients making decisions about taking novel or alternative medicines described the process as highly individualised [31]. The acknowledgement of these feelings is likely important in empowering patients, and does appear to be effective and well-received. Qualitative interviews in a trial of CBT for anxiety in advanced cancer reported that patients perceived the approach as relevant and useful [32]. Overall, the exploration of PAT was considered an expansion of treatment approaches that acknowledged the varied needs of patients, and continuing to increase the repertoire of available treatments was valued.

### Balancing safety and risk

A general acceptance to the concept of PAT was also tempered with a desire to minimise risk. This was evident in the view that microdosing was preferred to larger doses. Favouring a conservative approach to dosing suggests patients and carers held caution with the idea of unfamiliar treatments. This suggests that while there may be an increasing openness to risky or unfamiliar therapies in the context of life-threatening illness, this openness still has its limits.

Participants also had conflicting views about natural versus synthetic compounds. Many people express a preference for natural medicines and foods relative to synthetic alternatives [33] and, similarly, participants commonly framed their more favourable perceptions of naturally occurring psychedelics as based on the view that natural seemed “less risky”. However, participants also reported desiring products which are trusted and have been subject to rigorous scientific testing. The common tie between these apparently opposing preferences is an orientation to cues of safety. While natural compounds seem to innately signal safety by appearing “gentler”, synthetic products demonstrate evidence of safety in the rigorous clinical testing involved in their production.

Participants’ perceptions of risk were often tied to perceptions of recreational drug use in the ‘60s and ‘70s. Individual knowledge and perceptions are coloured by wider societal attitudes [34]. Ultimately, this cultural knowledge appeared to inform concerns about psychedelics; for instance, that people were having bad trips or engaging in experimentation. Public attitudes about such concepts tend to be negatively skewed, and hence the potential stigma of associations between psychedelics and the Hippie era. Increasing representation of fact-based narratives on therapies using psychedelics may be helpful in overcoming misconceptions.

### Existing knowledge of cannabis as a heuristic

Reference to cannabis by participants in this study can be understood as a heuristic. The similarity heuristic describes how people make evaluations of ideas for which they have limited information, based on comparisons to a sufficiently similar concept or event [35]. The use of such a strategy by participants in this study suggests people make sense of novel treatments by referring to established ones. However, such processes are approximate and have a tendency to produce biased or uninformed judgements.

### Study limitations

As with all qualitative inquiry, this study is limited in its ability to generalise findings more broadly. This study was conducted in New Zealand and therefore may not be generalisable to cancer populations in other countries. Further, the sample included only two Indigenous (Māori) participants and, as such, is limited in being able to comment on this perspective. Given the inequities in health outcomes and care that are known to exist for Indigenous populations, this is an important concern. This is significant especially when considering the historical context of naturally occurring substances and their links to Indigenous cultural practices, particularly given that concerns about how such compounds are procured were voiced by one Māori participant. Beginning to test these therapies in clinical trials is a logical direction. Future PAT should involve spiritual and Māori perspectives of health [36]. Similarly, most participants identified as female, with only four male participants recruited. Finally, it is notable that very few negative reactions were reported. Although our study advertisements did not mention psychedelics in an attempt to minimise bias and our interview questioning specifically asked about concerns and negative views (as well as positive beliefs), it is possible that our positionality as researchers within a psychological medicine context may have resulted in bias.

## Conclusion

This work sought to examine and describe the knowledge, perceptions, beliefs and attitudes of advanced cancer patients and their carers regarding PAT. Knowledge appeared heavily informed by associations with recreational drugs, which at times coloured perceptions in negative ways. Despite this, reactions to the concept of psychedelic therapy were positive in most cases, ranging from relief to intrigue and excitement. Participants’ views on PAT demonstrated concern about safety while also endorsing anything that helps. This raises a concern that in the context of many countries where PAT is not available and most psychedelics are classified as Schedule 1 drugs, patients who are desperate for relief from their distress might self-source psychedelics for self-administration [24]. Researchers and clinicians should be aware of this possibility and the associated risks and be mindful of this in communications with this population. Nevertheless, as the research in this area continues to develop, allowing the perspectives of stakeholders in advanced cancer to guide such interventions constitutes a meaningful step in improving the lives of those living with advanced cancer.

## Data Availability

The dataset used and analysed during the current study is available from the corresponding author on reasonable request.
